# Comparing the current knowledge & understanding of vitreo-retinal conditions & associated practice: optometrists versus year one specialty ophthalmic trainees in the UK

**DOI:** 10.1038/s41433-023-02909-x

**Published:** 2023-12-27

**Authors:** S. Rehan, R. McPherson

**Affiliations:** 1 Specialty Registrar, Royal Gwent Hospital, Cardiff Road, Newport, Wales NP20 2UB UK; 2 Consultant Ophthalmic Surgeon, University Hospital of Wales, Cardiff, Wales CF14 4XW UK

**Keywords:** Education, Health services

Ophthalmology is the busiest outpatient-based specialty in hospital medicine in the UK and demand is predicted to rise [1]. This is coupled with an acknowledgment by the Royal College of Ophthalmologists (UK) that there is a ‘severe’ national shortage of ophthalmologists [2]. Over the past two decades optometrists have been championed as being the solution to this workforce issue which would potentially come with the added benefit of more patients being seen and retained in the primary care setting. Subsequently there are now a wide variety of extended role training pathways available for optometrists [3]. Interestingly optometrists already have well defined scopes of practice within the hospital eye service environment [4]. The training programmes for optometry and ophthalmology training are very different.

The time dedicated to Ophthalmology within medical school curricula in the UK is poor and declining with many students getting less than 1-week’s worth of exposure to the specialty [5]. In the post pandemic era many specialties including ophthalmology continue to be taught as interactive non patient contact clinical attachments [6]. An ophthalmology trainee therefore entering OST1 can conceivably have little (unless they choose to pursue elective placements or taster weeks or do an ophthalmology 4-month rotation during their foundation junior doctor training period) or no prior clinical ophthalmology experience. Vitreoretinal conditions are complex, and their management involves uncertainty. Inappropriate and unnecessary referrals (including those with high false rates) burden secondary care services in ophthalmology. Clinical exposure and educational opportunities impact on level of knowledge and skills gained [7]. Do knowledge and understanding of Vitreoretinal conditions differ between optometrists and OST1 in the UK and do these factors result in differences in clinical practice?

In the first of its kind, community-based optometrist encompassing all experience levels and Ophthalmic Specialty Trainees coming to the end of their first year of training were invited to participate in a survey which compromised of 15 question items pertaining to knowledge and understanding of Vitreo-Retinal conditions and associated practice. The themes of the questions included were: detecting signs, spot diagnoses, current practice methods including referrals and follow up.

**Fig. 1:**
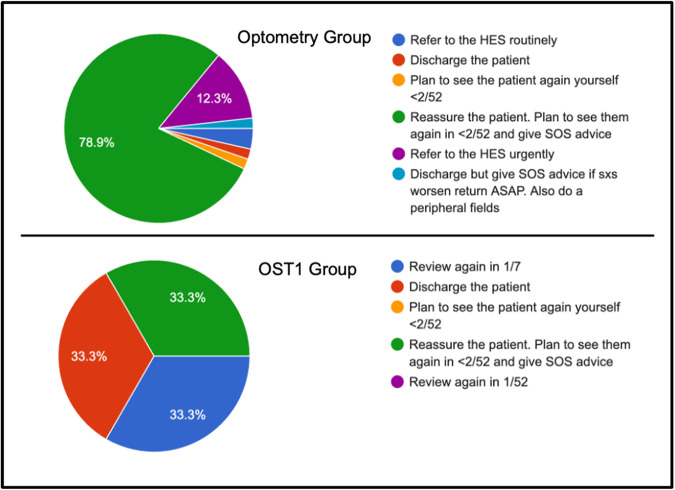
Showing the responses given to the question ‘A patient presents to you with flashing lights and floaters. On Examination (O/E) there is no tobacco dust, no breaks are seen, and no detachment is detected. The patient is very symptomatic. What would your next step be in the management of this patient?’

Significant differences were noted across all the themes assessed i.e. one question portrayed a fundal image of a retinal tear causing a retinal detachment. This was correctly diagnosed by 100% of the OST1 group compared to only 84.2% of optometry cohort. A staggering 56.1% of optometrists stated that they were ‘not confident at all’ identifying Shafer’s sign, which was not the case in the OST1 group (100% were ‘confident’). Skills to identity and differentiate chronic conditions such as chronic detachments, retinoschisis and macular holes were poor in both groups, more so in the optometry group. Other notable findings included: a) 20% of the optometry cohort do not dilate every patient presenting with flashing lights and floaters, b) only 14% of the optometry cohort use Tropicamide 1% and Phenylephrine 2.5% to dilate patients and c) in a symptomatic patient (flashing and floaters), with no detectable clinical signs, 12.3% of optometrists refer such patients to the hospital eye service (see Fig. 1). Interestingly, the majority of optometrists in our cohort, 73.7%, reported that they had 10 or more years of clinical experience.

**Fig. 2:**
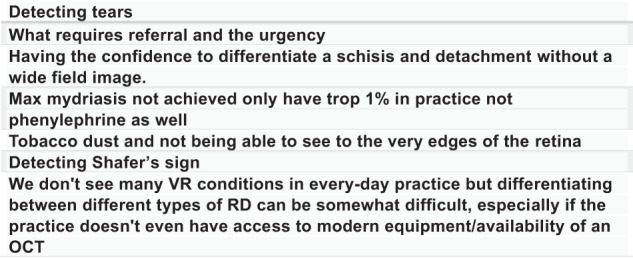
Demonstrating a selection of the aspects of the VR sub-speciality that optometry responders indicated that they struggle with. The accompanying question asked: ‘In your day-to-day practice, which aspects of the VR sub-speciality do you struggle with?’

A free text box response was also included at the end of the survey (for optometry responders, Fig. 2). A range of concerns were reported including detecting signs (tears and Shafer’s sign), inadequacy of dilatation achieved, differentiating between diagnoses and onwards referral and urgency.

Vitreoretinal pathologies (closely followed by corneal pathologies) are the most common reason for acute referral into the hospital eye service [8, 9]. However, in symptomatic posterior vitreous detachments the incidence rate of retinal tears is only 8.2% [10]. Despite there being generally low incidence rates for vitreoretinal pathologies, false positive referrals contribute significantly towards the volume of total referrals being made.

There is an intricate relationship between theory and practice and in combination they allow students to develop their knowledge and skills in order to provide optimal care. Therefore, if knowledge is lacking effective clinical performance cannot be achieved [11, 12]. In a paper from 2019, Hashemiparast and colleagues [13] very eloquently *state ‘Without mastery of theoretical knowledge, one cannot truly understand what he/she is doing and why they are using a particular procedure’*. From our data it can be broadly seen that the results obtained from the optometry group were inferior to those of the OST1 group. Many researchers have postulated as to why theory-practice gaps exists. Reasons include: resource restrictions, clinical experience, following the non-standard witnessed methods of staff in performing clinical practices, confidence issues, poor baseline knowledge and a lack of collaboration of clinical settings and educational institutions with students’ [11, 14, 15].

One must also consider that in the traditional non-specialized roles of community optometrists the major bulk of their work will entail seeing ‘normal’ patients and screening for pathology. This contrasts with the OST1 group who will primarily encounter patients with pathology. Moreover, this lack of exposure to pathology in the former group is a theme that begins and continues throughout their undergraduate optometry degrees. Later, historically as part of their pre-registration year optometrists would complete a short clinical attachment (i.e. 2-weeks) in the hospital eye care setting to increase their exposure to eye diseases. During the Covid-19 pandemic however such ‘in person’ attachments did not take place at all or alternatively virtual training modules were provided [16, 17]. When clinical exposure is lacking it can negatively impact on knowledge and confidence. An improved collaboration between educational institutions and clinical learning settings is needed.

In conclusion, our pilot project has identified and shown that poor knowledge, understanding coupled with inadequate examination (failing to dilate in the first instance/failing to use optimal dilating agents) can lead to poor/inappropriate referrals for both acute conditions (12.3% of optometrists refer symptomatic patients with no objective findings) and chronic pathology (10.5% of the optometry cohort believe that a macular hole always requires surgical intervention). The combination of confidence, knowledge and clinical experience contribute to effective clinical performance. Clear teaching and training needs have been identified.
